# Prediction of the Compressive Strength for Cement-Based Materials with Metakaolin Based on the Hybrid Machine Learning Method

**DOI:** 10.3390/ma15103500

**Published:** 2022-05-13

**Authors:** Jiandong Huang, Mengmeng Zhou, Hongwei Yuan, Mohanad Muayad Sabri Sabri, Xiang Li

**Affiliations:** 1 School of Mines, China University of Mining and Technology, Xuzhou 221116, China; huang@cumt.edu.cn (J.H.); ts21020232p21@cumt.edu.cn (M.Z.); ts21020224p21@cumt.edu.cn (X.L.); 2 Peter the Great St. Petersburg Polytechnic University, 195251 St. Petersburg, Russia; mohanad.m.sabri@gmail.com

**Keywords:** machine learning, compressive strength, random forests, firefly algorithm, hyperparameters

## Abstract

Cement-based materials are widely used in construction engineering because of their excellent properties. With the continuous improvement of the functional requirements of building infrastructure, the performance requirements of cement-based materials are becoming higher and higher. As an important property of cement-based materials, compressive strength is of great significance to its research. In this study, a Random Forests (RF) and Firefly Algorithm (FA) hybrid machine learning model was proposed to predict the compressive strength of metakaolin cement-based materials. The database containing five input parameters (cement grade, water to binder ratio, cement-sand ratio, metakaolin to binder ratio, and superplasticizer) based on 361 samples was employed for the prediction. In this model, FA was used to optimize the hyperparameters, and RF was used to predict the compressive strength of metakaolin cement-based materials. The reliability of the hybrid model was verified by comparing the predicted and actual values of the dataset. The importance of five variables was also evaluated, and the results showed the cement grade has the greatest influence on the compressive strength of metakaolin cement-based materials, followed by the water-binder ratio.

## 1. Introduction

Cement-based materials are composite materials that are composed of cement-based reinforcement, filler, chemical additives, and water through composite technology [1,2]. They are widely used in the construction industry because of their early strength, high strength, high mobility, strong durability, and other characteristics [3,4,5,6,7]. A large amount of CO_2_ is generated during the cement configuration process, which brings a great burden to the environment [8,9,10,11]. To reduce resource consumption and ease the burden of carbon emissions on the environment, researchers are looking into replacing some cement with active materials such as fly ash and silica fume [5,12,13,14,15,16]. Although the application of active materials is an effective method to reduce resource consumption and mitigate the greenhouse effect, due to the limited output of active materials, there are certain limitations in improving the performance of cement-based materials. Metakaolin, a kind of high-performance mineral admixture, is formed by calcination of kaolin at 600~800 °C. Metakaolin is rich in raw materials, has similar activity to silica fume, and has a better effect on improving the properties of cement-based materials [17,18,19,20].

At present, many scholars at home and abroad studied the application of metakaolin in cement-based materials and achieved abundant results. He et al. studied the influence of the content of metakaolin on the properties of sulfate cement-based materials [20]. The study showed that the initial fluidity, the expansion rate, the 28 d bending strength, and the bonding strength of sulfate cement-based materials were proportional to the content of metakaolin, while the compressive strength and the 7 d bending strength were in inverse proportion to the content of metakaolin. Yu et al. studied the influence of metakaolin on the frost resistance and the microstructure of the ceramsite concrete and concluded that metakaolin has a positive effect on improving the frost resistance of ceramsite concrete to some extent and that when the content of metakaolin is 10%, it has the best effect on improving the frost resistance of ceramsite concrete [21]. However, when the content is more than 20%, it has an adverse effect on the frost resistance of ceramsite concrete. Mo et al. studied the effect of metakaolin on the rheology and fiber distribution of ultra-high performance concrete (UHPC) [22]. The results showed that under the same fluidity, the apparent viscosity of UHPC increased with the increase in metakaolin content (5–10%), and there was no obvious relationship between the rheological properties and the content of metakaolin. The addition of metakaolin can improve the rate of slurry structure reconstruction, but increasing the amount of water-reducing agents has a weakening effect on this effect. In the static state, the reconstruction rate of the slurry structure is proportional to the dispersion effect of UHPC fiber. Qian et al. studied the influence of metakaolin content on the stress–strain relationship and bending strength of concrete [23]. The results show that the tensile strength, the bending strength, the compressive strength, and the peak strain are proportional to the content of metakaolin in a certain range, while the content of metakaolin has little effect on the tensile and compressive elastic modulus. Astutiningsih et al. studied the influence of Metakaolin Metastar and Metakaolin Bangka on the strength of ordinary cement [24]. Four metakaolin levels of 5%, 10%, 15%, and 20% were used in this investigation. The results show that both metakaolins can enhance the compressive strength of OPC, and the most appropriate strength can be obtained by replacing cement with 20% Metakaolin Metastar and 5% Metakaolin Bangka. There are many advantages to using metakaolin instead of cement to make cement-based materials, on the one hand, it can effectively improve the performance of cement-based materials, on the other hand, it can reduce the CO_2_ produced by cement manufacturing to a certain extent, and then relieve the pressure brought by carbon emissions to the environment. Therefore, using part of the metakaolin instead of cement to make cement-based materials has broad application prospects [25,26,27,28].

With the continuous improvement of the functional requirements of civil building infrastructure, the performance requirements of cement-based materials also tend to be diversified. In recent years, strength as an important index to evaluate the performance of cement-based materials has attracted more and more attention [7,29,30]. Typically, researchers test the strength of cement-based materials in the laboratory to find the right mix for the best strength of cement-based materials. However, the laboratory experiment method has many shortcomings, which need to spend a lot of time, energy, and money [31,32,33,34]. In order to solve this problem, a more efficient and economical method is needed to predict the performance of cement-based materials. Machine learning is an interdisciplinary subject involving many fields such as probability theory, statistics, and algorithm complexity theory [35,36]. Machine learning mainly studies how to simulate and implement human learning behavior, acquire new skills, and constantly improve their skills [37]. The essence of machine learning is to process and analyze data in large quantities through the computer’s powerful data processing and analysis ability [38,39]. In recent years, machine learning has been applied in many fields such as finance, medicine, education, and architecture due to its superior performance [40,41]. It can realize the automatic improvement of computer algorithms to simulate human learning, with data or experience and existing content knowledge structure classification, to effectively improve learning efficiency [42,43,44,45,46]. Machine learning is a common research hotspot in the field of artificial intelligence and pattern recognition. Because of its excellent performance, this method has been widely used to solve complex problems in engineering applications and scientific fields. In recent years, many researchers have proposed the method of machine learning to predict the properties of cement-based materials and achieved good results. Cheng et al. used an evolutionary LS-SVM model to predict the irritability of soil improvement based on micro-cement [47]. Guo et al. proposed an effective model for predicting the initial and final setting time of cement on a generalized learning system [48]. Yuanni et al. predicted the strength of concrete on the machine learning LGBM regression algorithm [49]. Fatih Ozcan et al. compared the prediction effect of neural network and fuzzy logic model on the long-term compressive strength of silica fume concrete [50]. Cheng Yeh used a neural network model to simulate the slump flow of concrete [51]. The above machine methods achieved good results in the performance prediction of cement-based materials, and machine learning methods were widely used in the prediction of cement-based materials [33,34,52,53,54,55,56,57,58,59]. Machine learning technology has been widely used in the cement-based materials performance evaluation process, but these methods still have some limitations, such as uncertainty, time-consuming, and low efficiency [60,61,62,63,64,65,66,67,68,69,70,71,72]. Therefore, it is necessary to propose a more efficient and simple machine learning technology to predict the compressive strength of metakaolin cement-based materials. A single machine learning model is difficult to solve the common shortcomings of machine learning models such as time-consuming and low efficiency [73,74,75,76,77,78,79,80]. To avoid the common problems of machine learning models and improve its application in the field of cement-based materials, the RF and FA hybrid machine learning model was proposed in this study. This hybrid model was employed for the prediction of the compressive strength of the cement-based materials with metakaolin. 

## 2. Methodology

### 2.1. Dataset Collection

Accurate prediction results are inseparable from an efficient evaluation model and reliable data. In previous studies, researchers focused more on developing more simple and efficient models to predict the properties of cement-based materials but often ignored the importance of a reliable database for predicting results. A database with reliable and sufficient data is the basis for verifying the accuracy of the model. In this study, the author collected the data from previous studies and established a large and reliable database as a dataset for predicting the compressive strength of cement-based materials with metakaolin. The specimen of the cement-based materials collected from the literature is the standard 150 mm size cube. In this database, the cement grade, the water to binder ratio, the binder to sand ratio, the metakaolin to binder ratio, and the superplasticizer were the input parameters, while the compressive strength of cement-based materials with metakaolin was the output parameter. The influence of these five parameters on the compressive strength of cement-based materials with metakaolin was confirmed in previous studies. Therefore, they were selected as the input variables in the present study; because compressive strength has been regarded as one of the most important parameters to evaluate the performance of cement-based materials, it was selected as the output variable. In the process of data collection, input variables are strictly screened: datasets containing five input variables (i.e., none of them is null) at the same time were selected. The database contains 361 datasets, which are randomly divided into the training set and test set (as shown in Appendix A, Table A1). The training set contains about 80% of the data, while the test set contains about 20% of the data.

### 2.2. RF and FA Hybrid Machine Learning Method

RF model has great advantages over other machine learning models, such as better performance, fast computing speed, strong anti-interference ability, and strong fitting ability [81,82]. However, the RF model is similar to a black box, and researchers cannot control its internal operation, so they can only try among different parameters and random seeds, which reduces the efficiency of and model operation to some extent [34,42]. In order to solve this problem, the optimal hyperparameters need to be determined before the RF model runs. Finding the optimal hyperparameter is a difficult task in machine learning. The performance of machine learning is directly related to the hyperparameter. The better the hyperparameter tuning is, the better the model running effect. In this study, the author used FA to tune the hyperparameter of the RF model. In other words, an RF and FA hybrid machine learning model was proposed to predict the compressive strength of cement-based materials in this study. In this hybrid machine learning model, FA is used to adjust the hyperparameters of the RF model, and the RF model is used to determine the complex nonlinear relationship between the compressive strength of metakaolin cement-based materials and the cement grade, the water to binder ratio, the binder to sand ratio, the metakaolin to binder ratio, the superplasticizer.

#### 2.2.1. Random Forest (RF) Model

RF is an integrated learning method that takes a decision tree as the basic unit and completes learning by integrating multiple decision trees. Intuitively speaking, RF is a classification method using decision trees as classifiers. For an input sample, n trees have n classification results; RF integrates all classification voting results and specifies the category with the largest number of votes as the final output. RF constructs multiple decision trees. In order to predict a sample, it is necessary to count the prediction results of each tree in the forest for the sample and then select the result with the highest vote as the final prediction result. The randomness of RF is reflected in the two aspects of random sampling, which make each decision tree in RF have the features of similarity and difference. RF construction includes randomly selected data and randomly selected eigenvalues to be selected.

In the machine learning process, the samples (which were named bootstrap sample $S_{n}^{\Theta }$) of the compressive strength was determined from the training dataset *S_n_* randomly. Hence, the probability regarding each sample should be 1/n. Afterward, the q bootstrap samples ($S_{n}^{\Theta_{1}},S_{n}^{\Theta_{2}},\ldots ,S_{n}^{\Theta_{q}}$) were assumed to be determined by employing the bagging algorithm to the dataset below.

(1) $$\hat{h}\left(X,S_{n}^{\Theta_{1}}\right),\hat{h}\left(X,S_{n}^{\Theta_{2}}\right),\ldots ,\hat{h}\left(X,S_{n}^{\Theta_{q}}\right)$$

in which the *q* output parameters ${\hat{Y}}_{1}=\hat{h}\left(X,S_{n}^{\Theta_{1}}\right),{\hat{Y}}_{2}=\hat{h}\left(X,S_{n}^{\Theta_{2}}\right),\ldots ,{\hat{Y}}_{q}=\hat{h}\left(X,S_{n}^{\Theta_{q}}\right)$ are determined from the *q* regression trees. Finally, the *q* output parameters should be averaged to determine the desired variable. The detailed process can be described as follows.

(i) Random sampling of data
  The random selection of data first involves sampling from the original dataset and constructing a sub-dataset with the same amount of data as the original data. Elements of different subsets and elements of the same subset can both be repeated. Then, the sub-decision tree is constructed by using the sub-dataset, and the input data are put into each sub-decision tree, and each sub-decision tree output a result. Finally, the data to be tested are put into each decision tree, and the output result of the random forest is obtained by voting the judgment result of the sub-decision tree.
(ii) Random selection of features to be selected
  Each split process of the random forest subtree only uses part of the features to be selected, which are randomly selected from all the features to be selected, and then the optimal feature is selected from the randomly selected features. Random selection of features to be selected can improve the diversity of the system and thus improve classification skills.

#### 2.2.2. Firefly Algorithm (FA)

FA works by treating each point in space as a firefly and completing the optimization process by taking advantage of the characteristic that fireflies with strong luminescence attract fireflies with weak luminescence [7,83]. The weak firefly moves to the strong firefly to complete position iteration, find the optimal position and complete the search process. FA needs to meet the following conditions:

(i) Suppose all fireflies are attracted to each other and of the same sex;
(ii) The attraction between fireflies is only related to luminous intensity and location. The strong fireflies move randomly and attract the weak fireflies around, and the attraction is inversely proportional to the distance between fireflies;
(iii) Luminescence intensity is determined by the objective function and is proportional to the specified function in the specified region. The search process is related to the luminance and mutual attraction of fireflies, and these two parameters are inversely proportional to the distance. The brighter the firefly is, the better its position is, and the brightest firefly represents the optimal solution for the function. The brighter the firefly is, the more attracted it is to the surrounding fireflies, and if the fireflies glow at the same intensity, they move randomly.

## 3. Results and Discussion

### 3.1. Correlation Analysis

**Figure 1:**
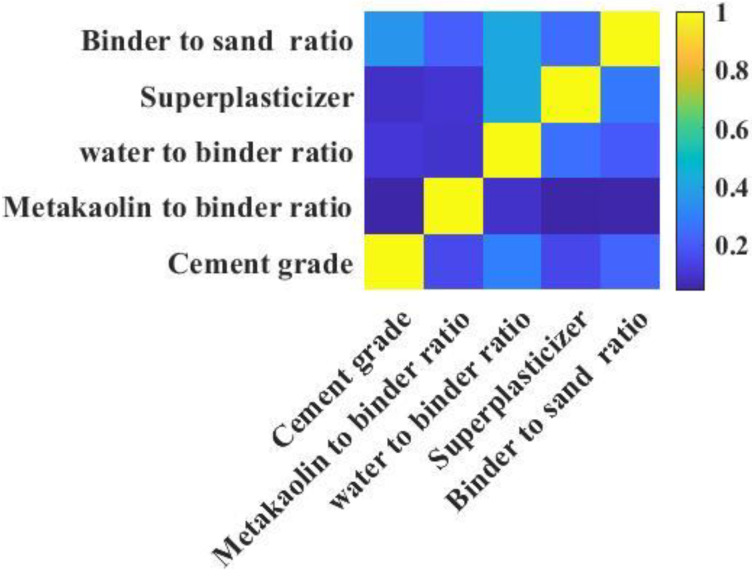
Correlation coefficients matrix diagram.

Correlation analysis refers to the analysis of two or more correlated variables to measure the degree of closeness between variables through the analysis results. The high correlation between input parameters means that the correlation coefficient is a high negative value or high positive value, which may lead to low efficiency of the model or difficulty to explain the influence of input parameters on output parameters. Therefore, before training with the RF model, the correlation between cement grade, metakaolin to binder ratio, water to binder ratio, superplasticizer, and binder to sand ratio should be analyzed first. In this study, the author used Statistical Product Service Solutions (SPASS) to analyze the correlation between input parameters, and the analysis results are shown in Figure 1. Figure 1 shows that the correlation coefficient between the same input parameters is 1. The correlation between water to binder ratio and superplasticizer, water to binder ratio and binder to sand ratio, cement grade and binder to sand ratio was the highest, with a correlation coefficient of 0.5, while the correlation between metakaolin to sand ratio and cement grade, metakaolin to binder ratio and superplasticizer, metakaolin to binder ratio and binder to sand ratio was the lowest, with a correlation coefficient of 0.2. In summary, the correlation coefficients between the five input parameters were all lower than 0.6, indicating these parameters were independent of each other. Therefore, there is no multicollinear problem as the cement grade, metakaolin to binder ratio, water to binder ratio, superplasticizer, and cement to sand ratio were employed as the input parameters to predict the compressive strength of cement-based materials with metakaolin.

### 3.2. Correlation Coefficients Matrix Diagram

**Figure 2:**
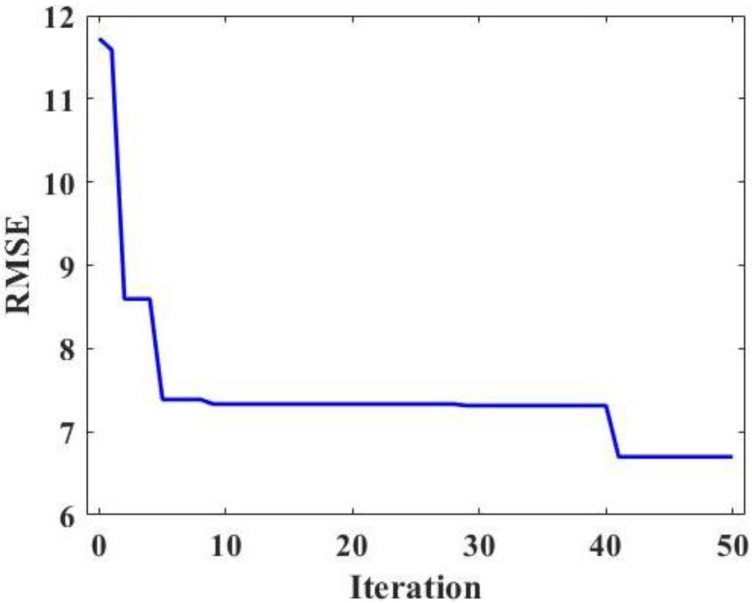
Relationship between the iteration and RMSE.

Hyperparameters are parameter values set before the machine learning process, not parameter data found through training. Hyperparameter tuning refers to the optimization of hyperparameters and the selection of a group of optimal hyperparameters for machine learning. Therefore, hyperparameter tuning plays an important role in improving the performance and efficiency of machine learning. In this study, the FA algorithm is used to tune the hyperparameter of the RF model. In order to select the optimal hyperparameters, 50 iterations were carried out in this study, and the relationship between the RMSE value and iteration times is shown in Figure 2. It can be seen clearly from Figure 2 that the RMSE value decreases sharply at first and then tend to be stable with the increase in iterations, proving that the FA algorithm can effectively adjust the hyperparameters of the RF model. Before the 10th iteration, the minimum RMSE value was obtained, and then with the increase in iterations, the RMSE value tends to be stable. Therefore, 10-fold cross-validation was used to obtain the optimal hyperparameters.

**Figure 3:**
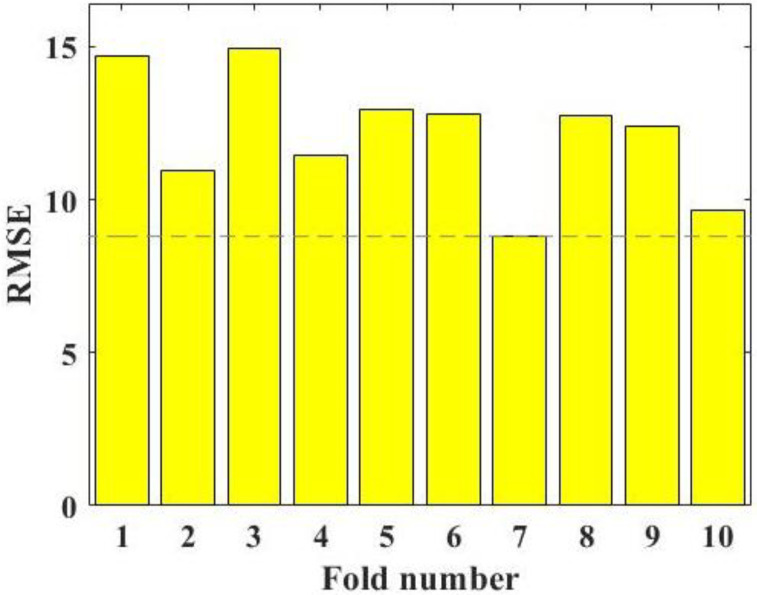
RMSE results of the hyperparameter tuning.

Ten-fold cross-validation means that the training sets are divided into 10 groups, one group is selected as the test set in turn, the remaining 9 groups are selected as the training set, and the optimal hyperparameter is selected through the results 10 times. Before using machine learning models to predict the compressive strength of metakaolin cement-based materials, using 10-fold cross-validation for hyperparameter tuning can effectively avoid over-learning or under-learning state and improve the reliability of the final prediction results of the model. RMSE values of the RF model with different folds are shown in Figure 3. Figure 3 shows that the minimum RMSE value of the RF model is obtained at the 7th fold; that is, selecting this value as the optimal hyperparameter of the RF model can make the prediction results of the final model more persuasive.

### 3.3. Model Evaluation

**Figure 4:**
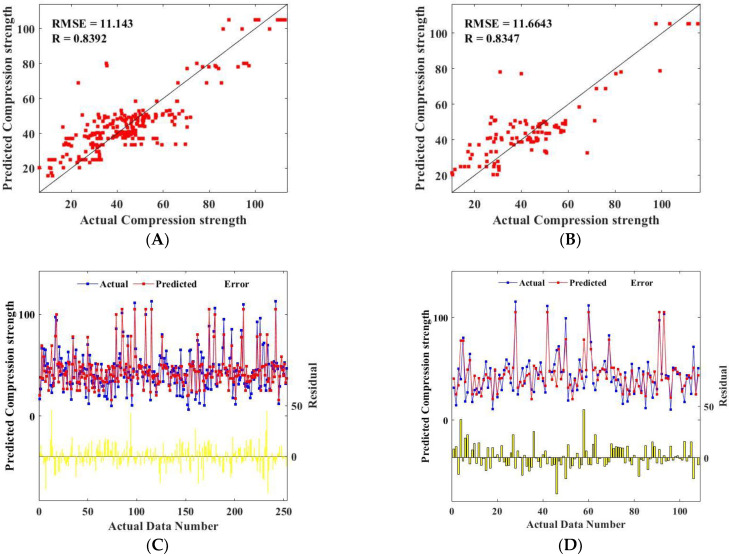
Comparison of the actual compressive strength and predicted compressive strength (RF model): (**A**) predicted results of the training set; (**B**) predicted results of the testing set; (**C**) comparison results of the training set; (**D**) comparison results of the testing set.

After the RF and FA hybrid machine learning model is established to predict the compressive strength of metakaolin cement-based materials, it is very important to evaluate the model. The evaluation results determine whether the model has practical value, which is whether the model can accurately predict the compressive strength of metakaolin cement-based materials. This study evaluated the accuracy of the model by comparing the predicted and actual values of the training set and the test set. The prediction results of the compressive strength training dataset and test dataset of metakaolin cement-based materials are shown in Figure 4.

Figure 4A shows the predicted results of the training set, and Figure 4B shows the predicted results of the test set. As can be seen from Figure 4A, most of the actual and predicted values of compressive strength of the training set are concentrated in 20–60 MPa, and a few are concentrated in 60–115 MPa. The actual maximum and minimum values of compressive strength in the training set were 113 MPa and 6 MPa, respectively, and the predicted values were 108 MPa and 20 MPa, respectively. The actual value corresponding to the point with the largest deviation in the training set is about 40 MPa, and the corresponding predicted value is about 80 MPa. As can be seen from Figure 4B, the actual and predicted values of the compressive strength of the test bench are mostly 20–60 MPa, and a small part is 60–115 MPa. The actual maximum and minimum compressive strength of the test bench is 112 MPa and 9 MPa, respectively, and the predicted values are 108 MPa and 21 MPa, respectively. The predicted value corresponding to the maximum deviation point in the training set is about 78 MPa, while the actual value is about 33 MPa. In general, the predicted values of the training set and test set are basically consistent with the actual values, but there are some points where the predicted values differ greatly from the actual values. Meanwhile, by comparing the predicted results of the training set and the test set, it is found that the R values of both are higher (0.8392 and 0.8347), and the RMSE values are lower (11.143 and 11.6643). Considering the 361 databases come from different studies. Therefore, there are great differences regarding the raw materials in morphology characteristics, chemical composition, and other factors. Hence, the RF-FA mixed machine learning model proposed in this study can be used to predict the compressive strength of metakaolin cement-based materials, and the predicted value is in good agreement with the measured value; thus, this method can accurately and effectively predict the compressive strength of metakaolin cement-based materials.

The comparison between predicted and measured compressive strength values of the training set and test set of metakaolin cement-based materials is shown in Figure 4. The horizontal line in the figure represents the difference between the predicted value of compressive strength and the actual value. As shown in Figure 4C,D, the predicted values of compressive strength in the training set and test set have a high consistency with the actual values but fewer error points. This proves once again that the hybrid machine learning model has a good effect on the prediction of compressive strength of metakaolin cement-based materials.

### 3.4. Variable Importance Evaluation

**Figure 5:**
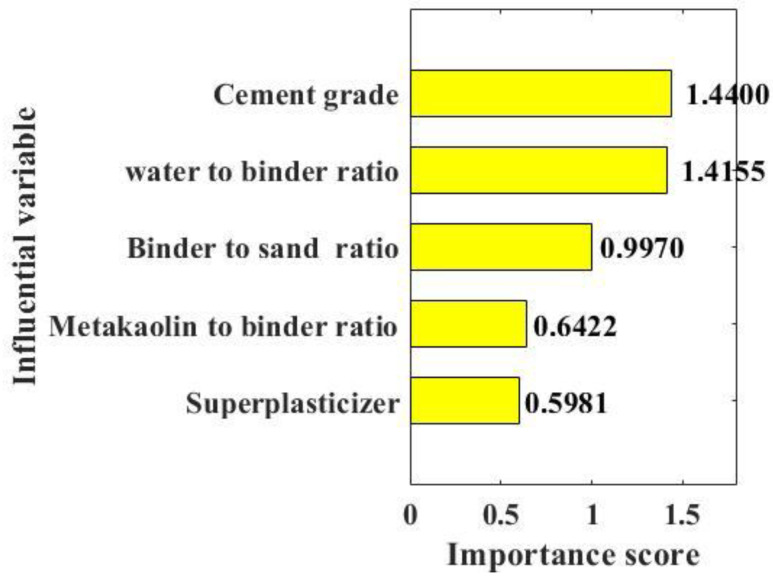
Variable importance of the compressive strength.

The above analysis shows that RF and FA hybrid machine learning method provides an efficient and simple prediction method for the compressive strength of metakaolin cement-based materials. It is of great practical significance to determine the importance of cement grade, metakaolin to binder ratio, water to binder ratio, superplasticizer, and binder to sand ratio on the compressive strength of metakaolin cement-based materials. In this study, the machine learning method was used to determine the importance of these five input parameters to the compressive strength of metakaolin cement-based materials, and the results are shown in Figure 5. As can be seen from Figure 5, the influence scores of cement grade, metakaolin to binder ratio, water to binder ratio, superplasticizer, and binder to sand ratio on the compressive strength of metakaolin cement-based materials are 1.4400, 1.4155, 0.9970, 0.6422 and 0.5981, respectively, the degree of influence decreases one by one. The influence scores of the five input parameters on the compressive strength of metakaolin cement-based materials are all positive; thus, the compressive strength of metakaolin cement-based materials increases with the increase in any one of the five parameters and decreases with the decrease in any one of the five parameters. The most important factor affecting the compressive strength of metakaolin cement-based materials is cement grade, followed by water to binder ratio, while the superplasticizer has the least influence on the compressive strength of metakaolin cement-based materials. The analysis of the importance of the cement grade, the water to binder ratio, the binder to sand ratio, the metakaolin to binder ratio, the superplasticizer on the compressive strength of metakaolin cement-based materials can provide some references for engineers in designing metakaolin cement-based materials with high compressive strength. In order to obtain a higher compressive strength of cement-based materials with metakaolin, the engineers can pay more attention to the cement grade and the water to binder ratio when designing the mixture ratio of the cement-based materials. However, the content of the superplasticizer can be paid less attention considering its little influence on the compressive strength.

## 4. Conclusions

In order to solve the shortcomings of traditional machine learning models in cement-based material performance prediction, such as uncertainty, low time consumption, and low efficiency, and improve the accuracy of model prediction, this paper proposes an RF and FA hybrid machine learning model to predict the compressive strength of metakaolin cement-based materials. The accuracy of the hybrid machine learning model is verified by comparing the predicted and actual values of the training set and the test set. The following conclusions are drawn:

- Through correlation analysis, it is found that the correlation coefficient of cement grade, the proportion of water binder, the ratio of binder sand, the proportion of metakaolin binder, and the efficient, reducing agent are all less than 0.6, and these five parameters are independent of each other. Therefore, using these five parameters as input parameters to predict the compressive strength of metakaolin cement-based materials will not appear multicollinearity;
- The results of 50 iterations show that RMSE decreases sharply with the increase in iterations and then tends to be basically stable. Therefore, using the FA model to adjust the hyperparameters of the RF model can achieve desired results. RF and FA hybrid machine learning algorithms were used to predict the compressive strength of metakaolin cement-based materials, and the training set and test set between predicted values and measured values had a high consistency (RMSE of the training and testing datasets are 11.143 and 11.6643, respectively; R of the training and testing datasets are 0.8392 and 0.8347, respectively), indicating the hybrid model can accurately predict the compressive strength of metakaolin cement-based materials;
- Among the five input variables (cement grade, water-binder ratio, cement-sand ratio, metakaolin ratio, and high-efficiency water-reducing agent), cement grade has the greatest influence on the compressive strength of metakaolin cement-based materials, followed by the water-binder ratio. High-efficiency water reducing agent has the least effect. Therefore, cement gradation and water-binder ratio should be mainly considered in the mix design of metakaolin cement-based materials.

For future development, a comparative study should be carried out based on different algorithms from the perspectives of computing efficiency, reliability, and accuracy. Moreover, more possible data on cement-based materials with metakaolin should be collected to increase the reliability of the prediction model.

## Data Availability

The data presented in this study are available on request from the corresponding author.

## Appendix A

**Table A1:** Dataset used in the present research.

Cement Grade	Metakaolin to Binder Ratio	Water to Binder Ratio	Superplasticizer	Binder to Sand Ratio	Compression Strength
53	0	0.5	0	0.33	17.6
53	0	0.5	0	0.33	15.8
53	0	0.5	0	0.33	14.2
53	0	0.5	0	0.33	12.8
53	0	0.5	0	0.33	11.8
53	0	0.5	0	0.33	10.4
53	0	0.5	0	0.33	24.8
53	0	0.5	0	0.33	25
53	0	0.5	0	0.33	25.2
53	0	0.5	0	0.33	24.8
53	0	0.5	0	0.33	22.2
53	0	0.5	0	0.33	19.6
53	0	0.5	0	0.33	29.2
53	0	0.5	0	0.33	29.6
53	0	0.5	0	0.33	30.2
53	0	0.5	0	0.33	29.4
53	0	0.5	0	0.33	26.4
53	0	0.5	0	0.33	23.4
53	0	0.5	0	0.33	30.6
53	0	0.5	0	0.33	31.8
53	0	0.5	0	0.33	33
53	0	0.5	0	0.33	31
53	0	0.5	0	0.33	28.4
53	0	0.5	0	0.33	25.4
42.5	0	0.5	0.8	0.44	33.4
42.5	0	0.5	0.8	0.44	19.1
42.5	0	0.5	0.8	0.44	16.8
42.5	0	0.5	5	0.44	16.3
42.5	0	0.5	0.8	0.44	18.9
42.5	0	0.5	1.6	0.44	17.8
42.5	0	0.5	0.8	0.44	17.4
42.5	0	0.5	1.6	0.44	18.1
42.5	0	0.5	2	0.44	22
42.5	0	0.5	1.6	0.44	20.5
42.5	0	0.5	1.6	0.44	19.7
42.5	0	0.5	1.6	0.44	19.4
42.5	0	0.5	0.8	0.44	46.3
42.5	0	0.5	0.8	0.44	31.1
42.5	0	0.5	0.8	0.44	27
42.5	0	0.5	5	0.44	34.5
42.5	0	0.5	0.8	0.44	30.6
42.5	0	0.5	1.6	0.44	36.9
42.5	0	0.5	0.8	0.44	29.3
42.5	0	0.5	1.6	0.44	31.8
42.5	0	0.5	2	0.44	36.1
42.5	0	0.5	1.6	0.44	31.2
42.5	0	0.5	1.6	0.44	32.7
42.5	0	0.5	1.6	0.44	33.1
42.5	0	0.5	0.8	0.44	59.4
42.5	0	0.5	0.8	0.44	50.5
42.5	0	0.5	0.8	0.44	33.7
42.5	0	0.5	5	0.44	70.3
42.5	0	0.5	0.8	0.44	47.7
42.5	0	0.5	1.6	0.44	55.6
42.5	0	0.5	0.8	0.44	43.4
42.5	0	0.5	1.6	0.44	51.3
42.5	0	0.5	2	0.44	57.2
42.5	0	0.5	1.6	0.44	46.8
42.5	0	0.5	1.6	0.44	51
42.5	0	0.5	1.6	0.44	49.4
52.5	0	0.6	0	0.33	17.6
52.5	0	0.6	0	0.33	29.4
52.5	0	0.6	0	0.33	44.5
52.5	0	0.6	0	0.33	57.5
52.5	20	0.6	0	0.33	18.8
52.5	20	0.6	0	0.33	29.1
52.5	20	0.6	0	0.33	50.4
52.5	20	0.6	0	0.33	69.7
52.5	10	0.6	0	0.33	20
52.5	10	0.6	0	0.33	32
52.5	10	0.6	0	0.33	50.7
52.5	10	0.6	0	0.33	68.1
52.5	20	0.6	0	0.33	18
52.5	20	0.6	0	0.33	28.5
52.5	20	0.6	0	0.33	50
52.5	20	0.6	0	0.33	65.3
52.5	0	0.36	1.4	0.5	40
52.5	0	0.36	1.4	0.5	70.42
52.5	0	0.36	1.4	0.5	80.42
52.5	0	0.36	1.4	0.5	84.17
52.5	10	0.36	2.35	0.5	35.21
52.5	10	0.36	2.35	0.5	74.58
52.5	10	0.36	2.35	0.5	95
52.5	10	0.36	2.35	0.5	96.25
52.5	10	0.36	2.04	0.5	35.63
52.5	10	0.36	2.04	0.5	82.71
52.5	10	0.36	2.04	0.5	97.29
52.5	10	0.36	2.04	0.5	99.17
32	0	0.48	0	0.36	16.56
32	10	0.48	0	0.36	18.99
32	15	0.5	0	0.36	18.17
32	20	0.51	0	0.36	18.13
32	25	0.52	0	0.36	16.3
32	30	0.53	0	0.36	15.45
32	0	0.53	0	0.5	25.5
32	10	0.53	0	0.5	25.9
32	15	0.53	0	0.5	28.4
32	20	0.53	0	0.5	28.2
32	25	0.53	0	0.5	27.5
32	30	0.53	0	0.5	26.8
32	0	0.5	0	0.5	29.3
32	10	0.5	0	0.5	30.4
32	15	0.5	0	0.5	31.3
32	20	0.5	0	0.5	29.4
32	25	0.5	0	0.5	29.1
32	30	0.5	0	0.5	26.8
32	0	0.47	0	0.5	31.5
32	10	0.47	0	0.5	31.9
32	15	0.47	0	0.5	32.6
32	20	0.47	0	0.5	30.4
32	25	0.47	0	0.5	30.2
32	30	0.47	0	0.5	29
32	0	0.44	0.5	0.5	35.4
32	10	0.44	0.5	0.5	38
32	15	0.44	0.5	0.5	36.6
32	20	0.44	0.5	0.5	35.7
32	25	0.44	0.5	0.5	33.8
32	30	0.44	0.5	0.5	31.7
32	0	0.4	1.3	0.5	40
32	10	0.4	1.3	0.5	42.4
32	15	0.4	1.3	0.5	41.9
32	20	0.4	1.3	0.5	41.4
32	25	0.4	1.3	0.5	39.6
32	30	0.4	1.3	0.5	35.5
32	0	0.48	0	0.36	23.68
32	10	0.48	0	0.36	25.19
32	15	0.5	0	0.36	26.24
32	20	0.51	0	0.36	25.42
32	25	0.52	0	0.36	23.25
32	30	0.53	0	0.36	22.48
32	0	0.53	0	0.5	36.2
32	10	0.53	0	0.5	38.5
32	15	0.53	0	0.5	40
32	20	0.53	0	0.5	40.2
32	25	0.53	0	0.5	38.1
32	30	0.53	0	0.5	35.7
32	0	0.5	0	0.5	38.3
32	10	0.5	0	0.5	41.3
32	15	0.5	0	0.5	42.1
32	20	0.5	0	0.5	42.7
32	25	0.5	0	0.5	39.9
32	30	0.5	0	0.5	38.1
32	0	0.47	0	0.5	39.5
32	10	0.47	0	0.5	43.6
32	15	0.47	0	0.5	44.1
32	20	0.47	0	0.5	44
32	25	0.47	0	0.5	42.8
32	30	0.47	0	0.5	38.7
32	0	0.44	0.5	0.5	41.8
32	10	0.44	0.5	0.5	45.9
32	15	0.44	0.5	0.5	46.3
32	20	0.44	0.5	0.5	45.4
32	25	0.44	0.5	0.5	44.7
32	30	0.44	0.5	0.5	40.6
32	0	0.4	1.3	0.5	44
32	10	0.4	1.3	0.5	47.2
32	15	0.4	1.3	0.5	48.1
32	20	0.4	1.3	0.5	49
32	25	0.4	1.3	0.5	47.7
32	30	0.4	1.3	0.5	42.1
32	0	0.48	0	0.36	28.16
32	10	0.48	0	0.36	29.2
32	15	0.5	0	0.36	30.94
32	20	0.51	0	0.36	29.98
32	25	0.52	0	0.36	28.45
32	30	0.53	0	0.36	27.97
32	0	0.53	0	0.5	41.3
32	10	0.53	0	0.5	43.8
32	15	0.53	0	0.5	43.4
32	20	0.53	0	0.5	44.3
32	25	0.53	0	0.5	42.5
32	30	0.53	0	0.5	39.6
32	0	0.5	0	0.5	44.5
32	10	0.5	0	0.5	46
32	15	0.5	0	0.5	44.7
32	20	0.5	0	0.5	45.1
32	25	0.5	0	0.5	43.6
32	30	0.5	0	0.5	42.1
32	0	0.47	0	0.5	46.3
32	10	0.47	0	0.5	46.9
32	15	0.47	0	0.5	45.2
32	20	0.47	0	0.5	47
32	25	0.47	0	0.5	43.9
32	30	0.47	0	0.5	43.6
32	0	0.44	0.5	0.5	46.6
32	10	0.44	0.5	0.5	48.5
32	15	0.44	0.5	0.5	47.8
32	20	0.44	0.5	0.5	48.7
32	25	0.44	0.5	0.5	47.3
32	30	0.44	0.5	0.5	43.9
32	0	0.4	1.3	0.5	48.7
32	10	0.4	1.3	0.5	50.9
32	15	0.4	1.3	0.5	51.1
32	20	0.4	1.3	0.5	52
32	25	0.4	1.3	0.5	51.3
32	30	0.4	1.3	0.5	47.5
32	0	0.48	0	0.36	29.74
32	10	0.48	0	0.36	31.23
32	15	0.5	0	0.36	32.1
32	20	0.51	0	0.36	32.16
32	25	0.52	0	0.36	32.28
32	30	0.53	0	0.36	30.4
32	0	0.53	0	0.5	43.7
32	10	0.53	0	0.5	44.5
32	15	0.53	0	0.5	45
32	20	0.53	0	0.5	44.2
32	25	0.53	0	0.5	43.4
32	30	0.53	0	0.5	43
32	0	0.5	0	0.5	47
32	10	0.5	0	0.5	46.7
32	15	0.5	0	0.5	46.9
32	20	0.5	0	0.5	45.3
32	25	0.5	0	0.5	45.3
32	30	0.5	0	0.5	44.9
32	0	0.47	0	0.5	48
32	10	0.47	0	0.5	47.4
32	15	0.47	0	0.5	47.4
32	20	0.47	0	0.5	46.8
32	25	0.47	0	0.5	45.6
32	30	0.47	0	0.5	45.1
32	0	0.44	0.5	0.5	50.5
32	10	0.44	0.5	0.5	51.2
32	15	0.44	0.5	0.5	51.4
32	20	0.44	0.5	0.5	50.6
32	25	0.44	0.5	0.5	50.5
32	30	0.44	0.5	0.5	47.1
32	0	0.4	1.3	0.5	51.4
32	10	0.4	1.3	0.5	54.2
32	15	0.4	1.3	0.5	54.9
32	20	0.4	1.3	0.5	53.8
32	25	0.4	1.3	0.5	53.5
32	30	0.4	1.3	0.5	50.8
42.5	0	0.5	0	0.33	34.5
42.5	5	0.5	0	0.33	34.4
42.5	10	0.5	0	0.33	32.4
42.5	15	0.5	0	0.33	31.7
42.5	20	0.5	0	0.33	27.4
42.5	0	0.5	0	0.33	47.1
42.5	5	0.5	0	0.33	46.3
42.5	10	0.5	0	0.33	48.6
42.5	15	0.5	0	0.33	47.9
42.5	20	0.5	0	0.33	49.4
42.5	0	0.5	0	0.33	49.7
42.5	5	0.5	0	0.33	57.5
42.5	10	0.5	0	0.33	58.8
42.5	15	0.5	0	0.33	63.8
42.5	20	0.5	0	0.33	61.5
42.5	0	0.5	0	0.33	57
42.5	5	0.5	0	0.33	65.1
42.5	10	0.5	0	0.33	70.2
42.5	15	0.5	0	0.33	71.2
42.5	20	0.5	0	0.33	68.4
52.5	0	0.45	0	0.33	47.94
52.5	0	0.45	0	0.33	57.2
52.5	0	0.45	0	0.33	66.23
52.5	0	0.45	0	0.33	64.6
52.5	0	0.45	0	0.33	66.01
42.5	0	0.55	0	0.4	40.08
42.5	5	0.55	0	0.4	44.79
42.5	10	0.55	0	0.4	56.76
42.5	15	0.55	0	0.4	56.58
42.5	0	0.49	0	0.36	37.27
42.5	0	0.49	0	0.36	28.53
42.5	10	0.49	0	0.36	38.22
42.5	0	0.49	0	0.36	41.75
42.5	0	0.49	0	0.36	50.05
42.5	0	0.49	0	0.36	39.05
42.5	10	0.49	0	0.36	48.9
42.5	0	0.49	0	0.36	52.83
42.5	0	0.49	0	0.36	56.76
42.5	0	0.49	0	0.36	52.56
42.5	10	0.49	0	0.36	54.76
42.5	0	0.49	0	0.36	58.87
32	0	0.48	0	0.36	6.06
32	0	0.48	0	0.36	10.49
32	0	0.48	0	0.36	10.73
52.5	0	0.33	0.03	0.51	85.99
52.5	0	0.33	0.03	0.51	94.3
52.5	0	0.33	0.03	0.51	106.28
52.5	15.15	0.33	0.03	0.51	100.15
52.5	15.15	0.33	0.03	0.51	111.29
52.5	15.15	0.33	0.03	0.51	112.99
52.5	15.15	0.33	0.03	0.51	101.66
52.5	15.15	0.33	0.03	0.51	109.7
52.5	15.15	0.33	0.03	0.51	112.87
52.5	15.15	0.33	0.03	0.51	97.38
52.5	15.15	0.33	0.03	0.51	111.17
52.5	15.15	0.33	0.03	0.51	115.25
52.5	15.15	0.33	0.03	0.51	88.61
52.5	15.15	0.33	0.03	0.51	103.27
52.5	15.15	0.33	0.03	0.51	111.72
52.5	0	0.5	0	0.33	24.8
52.5	0	0.5	0	0.33	49.5
52.5	0	0.5	0	0.33	59.8
52.5	0	0.5	0	0.33	62.8
43	0	0.4	0	0.33	26.37
43	0	0.4	0	0.33	43.13
43	0	0.4	0	0.33	46.7
43	0	0.4	0	0.33	48.08
42.5	0	0.6	0	0.33	10.43
42.5	10	0.6	0	0.33	11.22
42.5	15	0.6	0	0.33	11.44
42.5	20	0.6	0	0.33	11.66
42.5	25	0.6	0	0.33	11.78
42.5	30	0.6	0	0.33	9.75
42.5	0	0.4	0	0.33	22
42.5	5	0.4	0	0.33	31.5
42.5	10	0.4	0	0.33	30
42.5	15	0.4	0	0.33	29
42.5	20	0.4	0	0.33	27
42.5	0	0.4	0	0.33	32.5
42.5	5	0.4	0	0.33	40
42.5	10	0.4	0	0.33	43.5
42.5	15	0.4	0	0.33	42.1
42.5	20	0.4	0	0.33	44
42.5	0	0.4	0	0.33	40
42.5	5	0.4	0	0.33	52
42.5	10	0.4	0	0.33	56
42.5	15	0.4	0	0.33	60
42.5	20	0.4	0	0.33	58
42.5	0	0.4	0	0.33	48
42.5	5	0.4	0	0.33	52
42.5	10	0.4	0	0.33	64
42.5	15	0.4	0	0.33	67
42.5	20	0.4	0	0.33	65
52.5	0	0.3	0.01	0.33	31.1
52.5	0	0.3	0.01	0.33	77.24
52.5	0	0.3	0.01	0.33	82.45
52.5	0	0.3	0.01	0.33	92.5
52.5	0	0.3	0.01	0.33	79.92
52.5	0	0.3	0.01	0.33	83.06
52.5	10	0.3	0.01	0.33	22.99
52.5	10	0.3	0.01	0.33	66.37
52.5	10	0.3	0.01	0.33	75.9
52.5	10	0.3	0.01	0.33	85.25
52.5	10	0.3	0.01	0.33	71.98
52.5	10	0.3	0.01	0.33	78.75
42.5	0	0.45	0.5	0.5	37
42.5	0	0.45	0.5	0.5	48.6
42.5	0	0.45	0.5	0.5	56.8
42.5	5	0.45	0.5	0.5	33.8
42.5	5	0.45	0.5	0.5	46.3
42.5	5	0.45	0.5	0.5	55.5
42.5	10	0.45	0.4	0.5	43.2
42.5	10	0.45	0.4	0.5	50.4
42.5	10	0.45	0.4	0.5	56.9
42.5	15	0.45	0.4	0.5	41.8
42.5	15	0.45	0.4	0.5	51.2
42.5	15	0.45	0.4	0.5	63
42.5	0	0.3	0	0.62	34.5
42.5	0	0.3	0	0.62	47.1
42.5	0	0.3	0	0.62	49.7
42.5	0	0.3	0	0.62	57
42.5	0	0.3	0.7	0.76	36.07
42.5	0	0.3	0.7	0.76	26.83
42.5	0	0.3	0.7	0.76	44.91
42.5	0	0.3	0.7	0.76	32.14
42.5	0	0.3	0.7	0.76	65.02
42.5	0	0.3	0.7	0.76	45
42.5	0	0.3	0.7	0.76	71.94
42.5	0	0.3	0.7	0.76	48.33
